# A multi-centre stereotactic radiosurgery planning study of multiple brain metastases using isocentric linear accelerators with 5 and 2.5 mm width multi-leaf collimators, CyberKnife and Gamma Knife

**DOI:** 10.1093/bjro/tzae003

**Published:** 2024-01-30

**Authors:** Scott Hanvey, Philippa Hackett, Lucy Winch, Elizabeth Lim, Robin Laney, Liam Welsh

**Affiliations:** University Hospitals Plymouth NHS Trust, Plymouth, PL6 8DH, United Kingdom; The Royal Marsden, London, SW3 6JJ, United Kingdom; University Hospitals Bristol NHS Foundation Trust, Bristol, BS2 8ED, United Kingdom; University Hospitals Plymouth NHS Trust, Plymouth, PL6 8DH, United Kingdom; University of Plymouth, Plymouth, PL4 8AA, United Kingdom; University Hospitals Plymouth NHS Trust, Plymouth, PL6 8DH, United Kingdom; The Royal Marsden, London, SW3 6JJ, United Kingdom

**Keywords:** stereotactic radiosurgery, multi-leaf collimator, brain, metastases

## Abstract

**Objectives:**

This study compared plans of high definition (HD), 2.5 mm width multi-leaf collimator (MLC), to standard, 5 mm width, isocentric linear accelerator (linacs), CyberKnife (CK), and Gamma Knife (GK) for stereotactic radiosurgery (SRS) techniques on multiple brain metastases.

**Methods:**

Eleven patients undergoing SRS for multiple brain metastases were chosen. Targets and organs at risk (OARs) were delineated and optimized SRS plans were generated and compared.

**Results:**

The linacs delivered similar conformity index (CI) values, but the gradient index (GI) for HD MLCs was significantly lower (*P*-value <.001). Half the OARs received significantly lower dose using HD MLCs. CK delivered a significantly lower CI than HD MLC linac (*P*-value <.001), but a significantly higher GI (*P*-value <.001). CI was significantly improved with the HD MLC linac compared to GK (*P*-value = 4.591 × 10^−3^), however, GK delivered a significantly lower GI (*P*-value <.001). OAR dose sparing was similar for the HD MLC TL, CK, and GK.

**Conclusions:**

Comparing linacs for SRS, the preferred choice is HD MLCs. Similar results were achieved with the HD MLC linac, CK, or GK, with each delivering significant improvements in different aspects of plan quality.

**Advances in knowledge:**

This article is the first to compare HD and standard width MLC linac plans using a combination of single isocentre volumetric modulated arc therapy and multi-isocentric dynamic conformal arc plans as required, which is a more clinically relevant assessment. Furthermore, it compares these plans with CK and GK, assessing the relative merits of each technique.

## Introduction

Between 20% and 40% of all cancer patients will develop brain metastases.^1^ Therefore, the best treatment for this patient population presents a formidable challenge for any modern healthcare system, the patients and their families.

Surgical resection of brain metastases, particularly large tumours is paramount in improving local control.^2^ If surgery alone is used, recurrence is common. Whole brain radiotherapy (WBRT), delivered post-operatively, reduces the risk of recurrence to the surgical bed and incidence of new metastases.^3^^,^^4^ However, WBRT has not been demonstrated to improve overall survival outcomes and adverse consequences for the patient’s quality of life and cognitive function.^5^

Stereotactic radiosurgery (SRS) is increasingly used to deliver precise, high dose radiation to individual metastases as an alternative to WBRT,^6^ surgery and to irradiate resection cavities. The efficacy of SRS over WBRT post-operatively, has been substantiated in a randomized controlled trial. Furthermore, SRS has been shown to improve the quality of life and cognitive function for this cohort.^7^

Contemporary treatment planning algorithms automatically create single-isocentre, non-coplanar beam arrangements to optimize radiation dose delivery for isocentric linear accelerators (linacs). These isocentric conformal arc arrangements ensure radiotherapy plans deliver steep dose gradients to targets while minimizing dose to the normal tissue. Additionally, single isocentric treatments for multiple brain metastases can reduce treatment times, when compared with CyberKnife (CK; Accuray Inc., Sunnyvale, CA, United States) and multi-isocentric treatments like Gamma Knife (GK; Elekta AB, Stockholm, Sweden).^8^ A further advance in the linac-based SRS is the use of 6 degrees of freedom robotic couches which enable corrections for rotational set-up errors.^9^ Rotational errors can be corrected to within 0.5° for most patients using a robotic couch.^10^ Similarly, CK is equipped with a 6 degree of freedom robotic couch with sub-millimetre accuracy.^11^

Many studies have compared the plan quality of various treatment planning systems (TPSs) such as HyperArc™ (Varian Medical System, Palo Alto, CA, United States), Elements (Brainlab AG, Munich, Germany), Monaco HDRS (Elekta AB, Stockholm, Sweden) and treatment modalities such as GK and CK.^12–15^

A recent review article included a 5-case planning study, which compared plan quality from a linac equipped with high-definition multi-leaf collimators (HD MLCs), a linac with conical collimators, CK and GK. This study concluded that while comparable results could be achieved using any of these technologies, the disease specific characteristics have a greater influence on the appropriate patient selection than the technologies themselves.^16^

Another study found that linac and GK radiosurgery for the treatment of multiple brain metastases were associated with similar overall survival, while the incidence of radionecrosis was lower with linac-based radiosurgery compared with GK.^17^

The use of narrower MLC widths benefits Intensity Modulated Radiotherapy (IMRT) plan quality, particularly in the treatment of small targets.^18^^,^^19^ Improved target dose conformity and organ at risk (OAR) dose sparing has been demonstrated using 2.5 mm width MLCs, when compared to the more common 5 mm width MLCs for dynamic conformal arc (DCA) treatments of single targets.^20^ Another study compared 5 and 2.5 mm leaf width for a single isocentre multiple target volumetric modulated arc therapy (VMAT) delivery and concluded that the narrower MLCs delivered similar target dose conformity, with additional improvements in the low and moderate isodose spill.^21^ It might be expected that DCA plans would be more influenced by MLC width since it conforms to the target outline, as opposed to VMAT which defines MLC positions by inverse optimization.

While other studies have compared the plan quality of HD and standard width MLC linacs,^22–26^ these comparisons have kept the beam arrangements largely equivalent between plans being evaluated. To our knowledge, this multi-centre study is the first to compare plan quality of HD and standard width MLC linacs for a combination of both VMAT plans with a single isocentre per lesion and DCA plans using a single isocentre multiple target technique, which is a more clinically relevant assessment. The aims of this in silico study are to provide key information on the relative merits of HD MLC compared to standard MLC linacs, CK, and GK plans for stereotactic treatments.

## Methods

### Patient population

Eleven randomly selected patients, who had previously undergone SRS for a minimum of two metastatic brain tumours, who had originally undergone SRS using CK at the The Royal Marsden, London, were chosen for this retrospective planning study (62 targets in total). The number of lesions per patient ranged from 2 to 11. The mean volume of the targets was 1.02 ± 3.04 cm^3^ with a range of between 0.01 and 20.00 cm^3^. All patients had MRI scans reconstructed at 1 mm slice spacing for optimal tumour localization and delineation. The gross tumour volume (GTV) was delineated by the clinician using a contrast-enhanced MRI for all the patients. Table 1 details the target volumes and prescriptions for the 11 patients. Most of the targets (79%) were prescribed to 24 Gy, with a range of 18-24 Gy. The same delineated targets and critical organs were used across all modalities and the DICOM files were sent to the linac-based, CK, and GK centres to be planned on their TPS using the same prescription doses. All plans were prescribed to a single fraction, to be delivered in a single day, except for the first and third lesions of case 8, the seventh of case 10 and the first of case 11 all of which were prescribed to 3 consecutive daily fractions. Most of the targets were relatively spherical in shape, particularly the smaller (<1 cm^3^) structures.

**Table 1.** Target volumes and prescriptions of the 11 patients.

Target	Patient	1	2	3	4	5	6	7	8	9	10	11
GTV1	Px (Gy)	24	20	18	24	24	24	24	24	24	24	21
	Vol (cm^3^)	0.15	2.75	6.60	0.17	2.86	0.29	0.41	8.07	0.28	0.61	20.00
GTV2	Px (Gy)	24	20	24	24	24	24	24	24	24	20	24
	Vol (cm^3^)	0.06	0.35	0.10	0.10	0.22	0.31	0.03	0.07	0.25	0.05	0.07
GTV3	Px (Gy)	24	20	24	24	24	24	24	24	24	20	
	Vol (cm^3^)	0.03	0.34	1.55	0.02	0.53	0.19	0.02	2.70	0.15	0.04	
GTV4	Px (Gy)	24	20	24	24	24	24	24	24	24	20	
	Vol (cm^3^)	0.03	0.54	0.19	0.02	0.28	0.09	0.02	0.10	0.26	0.04	
GTV5	Px (Gy)	24	20				24	24		24	20	
	Vol (cm^3^)	0.02	0.16				0.08	0.15		0.01	0.02	
GTV6	Px (Gy)		24				24	24		24	20	
	Vol (cm^3^)		0.72				0.04	0.10		0.13	0.69	
GTV7	Px (Gy)						24	24		24	20	
	Vol (cm^3^)						0.02	0.03		0.04	10.10	
GTV8	Px (Gy)						24	24				
	Vol (cm^3^)						0.02	0.06				
GTV9	Px (Gy)							24				
	Vol (cm^3^)							0.03				
GTV10	Px (Gy)							24				
	Vol (cm^3^)							0.02				
GTV11	Px (Gy)							24				
	Vol (cm^3^)							0.04				
Overall Mean	Px (Gy)	23.15 ± 1.68										
	Vol (cm^3^)	1.02 (0-3.04)										

Abbreviations: GTV = Gross Tumour Volume, Px = prescription, Vol = target volume.

### Statistical analysis

The Shapiro-Wilk test was performed to determine whether the data were normally distributed using the R statistical package. The majority of the tests were not normally distributed; therefore, the Wilcoxon signed-rank test was employed to test statistical significance. A *P*-value threshold of less than .05 was used to define statistical significance.^27^ Statistically significant results are shown in italics in the tables.

### Planning techniques

A planning CT scan was used for the dose calculation of the linac plans and registered with an MRI scan as required for structure delineation guidance. Treatment plans were optimized using the original clinical dose prescription, which was chosen depending on tumour size and proximity to dose limiting OARs. The standard volume-based prescription dose and fractionations are based on data from the final report of the Radiation Therapy Oncology Group protocol.^28^ To minimize the risk of brain radionecrosis, it is preferable to use a prescription dose that will achieve an acceptably low dose to the adjacent normal brain tissue. For single fraction SRS, the V12Gy metric is used.^29^

For this study, no margins were applied to the GTV for consistency in comparing modalities. The optimization technique aimed to cover the entire target volume with the prescribed dose and minimized dose to surrounding healthy structures. The OARs considered were: the whole brain, brainstem, cerebellum, hippocampi, eyes, lenses, optic nerves, optic chiasm, optic tracts, and cochleae. Where the targets overlapped the OARs, only the dose to the remaining healthy tissue was reported. For bi-lateral OARs the highest dose was assessed. OAR dose tolerances were defined in accordance with Quantec^30^ and Emami.^31^

The linac plans were optimized with Elements treatment planning software using a combination of Multiple Brain Mets SRS or Cranial SRS, as required. VMAT plans with a dle isocentre per target were generated using Cranial SRS, while DCA plans treating multiple targets with a single isocentre were created with the Multiple Brain Mets SRS. These plans were first optimized for the Novalis Tx linear accelerator, equipped with HD MLCs, installed at the University Hospitals Plymouth NHS Trust. This linac has 60 leaf pairs, with a 2.5 mm width projection at isocentre for the inner 32 HD leaf and a 5 mm projection at isocentre for the outer 28 leaf pairs. Elements perform a pencil beam algorithm-based dose computation on the simulation CT images, with a dose grid resolution of 1 mm. The plans were calculated using a 6 MV SRS photon beam mode with a dose rate of 1000 monitor units (MU)/min. To ensure the treatments were optimized using the HD MLCs rather than the 5 mm width MLCs, the targets were examined from the beams eye view. A combination of plans with a single isocentre for multiple targets and single isocentre per lesion were created, ensuring all targets were treated with the HD MLCs. Therefore, the number of plans per patient varied depending on the distance of the lesions from each other. If a metastasis was located far from the other targets this may have to be treated with a single plan to cover the target with the HD MLCs as a single isocentre could not be used, the limiting factor being the maximum field size of 15 × 15 cm. The fewest possible number of plans, which covered the targets using the HD MLCs, were selected.

These plans were then optimized at The Royal Marsden, London for the (Varian 600C) with standard MLCs, which are 60 leaf pairs (5 mm projection at isocentre for the central 40 leaves and 10 mm projection at isocentre for the outer 20 leaves) using a pencil beam algorithm with a dose grid resolution of 1 mm and dose rate of 400MU/min and using a single isocentre for all plans.

**Figure 1.**
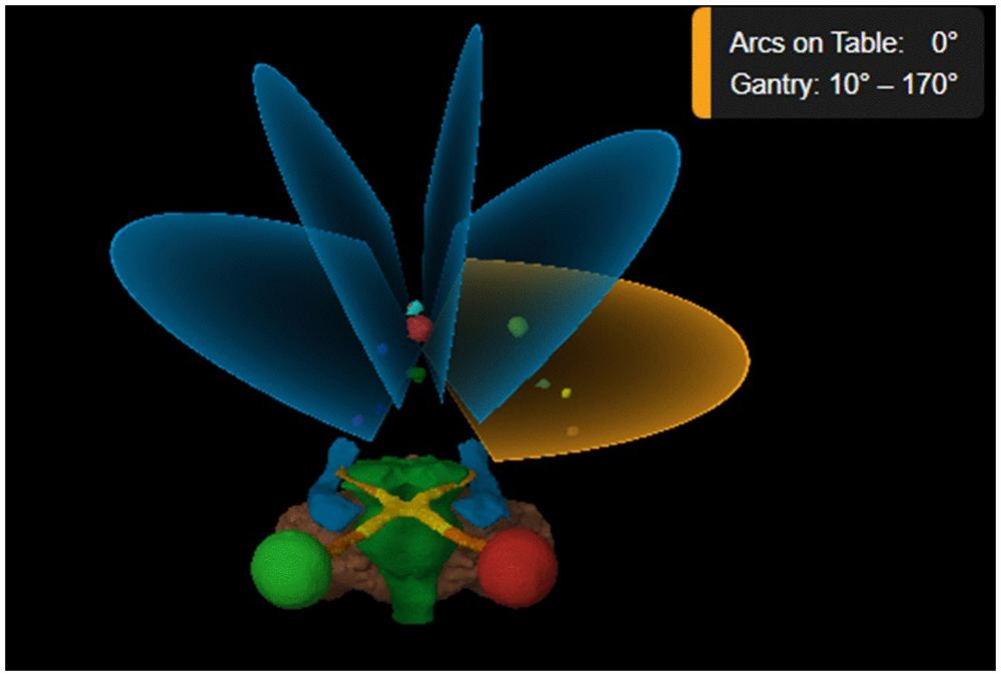
Five arc beam arrangement used in Elements Multiple Brain Mets SRS planning. OARs shown are left eye (red), right eye (green), optic nerve and optic tracts (orange), optic chiasm (yellow), brainstem (green), hippocampi (blue), and cerebellum (brown). Ten targets are shown in various colours throughout the arc arrangements.

For the linac plans a maximum of 6 bidirectional arcs were used. Intrafractional monitoring of isocentric position is achieved via serial orthogonal planar X-ray imaging. However, as this was a planning study, this was not required. The typical beam arrangement can be seen in Figure 1. The elements automatic planning algorithm determines the isocentre position as the average position of the centres of mass of each individual target. The software creates a set of 2 independent arcs per table angle using user defined table and gantry angles. The start and stop angles of the arc are first pre-defined, but are automatically adjusted as part of the optimization process. The MLC positions are conformed to the targets for all fields of every arc. Where multiple plans were required, the dose sum of all the plans per patient was used for analysis.

To minimize normal tissue irradiation all targets are assigned to specific arcs and may not be treated by all arcs at the same time. The algorithm aims to treat as many targets as possible by each arc, while ensuring the total number of MU is minimized.

The collimator angles are chosen by the software to distribute the dose spillage by radiation leakage between adjacent leaves. This rotation is limited by the fact that all targets must fit into the effective MLC field, but still minimizes dose to healthy tissue.

During optimization, the weights of arcs, position of the MLCs, and start and stop angles of each arc are modified by the TPS to improve dose conformity to targets and minimize dose to OARs.

The CK plans were optimized using a non-isocentric technique at The Royal Marsden, London on the Precision version 1.1.1.1 (Accuray Inc., United States) TPS with RayTracing algorithm, VOLO optimizer and dose grid resolution of 0.98 × 0.98 × 1 mm for delivery on a CK VSI System (Accuray Inc., United States) machine. The mean number of nodes per plan was 85 (ranging from 53 to 108) and mean number of beams was 247 (range 98-494). Fixed collimators were selected according to the size of each target with available sizes ranging from 5 to 60 mm. One fixed collimator was used for most single target treatments and up to 3 fixed collimators were used for multiple targets of varying sizes.

CyberKnife is a 6 MV linear accelerator mounted on a robotic arm with 6 degrees of freedom, combined with 6 degrees of freedom couch, delivering non-isocentric treatments. Orthogonal kV X-ray imaging is acquired throughout treatment, with a period that can be adjusted from 15 to 60 s. These kV images are compared against reference Digitally Reconstructed Radiographs (DRRs) in order to correct for intra-fraction movement. The use of intra-fractional monitoring was outside the scope of this study. A single metastasis treatment plan would typically consist of approximately 120 non-isocentric, non-coplanar beams, and would take about 30 min to deliver. As the number of metastases increases, the number of beams and the overall treatment time increase.^32^

Leksell Gamma Plan^®^ vs 11.1.1 was used for planning with GK in this study at the University Hospitals Bristol NHS Foundation Trust. This planning software uses a TMR10 algorithm in line with the Leksell Gamma Knife^®^ Icon™ functionality, where the tissue in the head is approximated to water. As such, a CT is not a pre-requisite for planning calculations and most outlining is performed on an MRI dataset. Treatment times are influenced by the daily dose rate which was around 2.849 Gy/min for this planning study. All plans were forward planned in accordance with local protocol to between 40% and 90% isodose of the prescribed dose, with larger lesions being partially planned utilizing an optimizer function.

The Leksell Gamma Knife^®^ Icon™ comprises 192 Co-60 sources mounted into 8 sectors (plates) in a conical fashion in the bore of the unit. These sectors slide superiorly and inferiorly over the surface of a multichannel collimation unit, so that each sector of 24 sources can line up with 4, 8, or 16 mm collimation holes, or be completely blocked. The collimated sources generate narrow beams that coincide at a single, central focus point in the radiation unit.^33^ Mechanical accuracy of this focus point is ensured by the manufacturer to be better than 0.4 mm and better than 0.5 mm over the entire range.^34^

Gamma Knife treatment can deliver both framed (Leksell G-frame) and frameless (masked-based). An X-ray arm that is mounted to GK unit is used to take a CBCT of the patient, in the treatment position and generate the 3D stereotactic co-ordinate system. A planning MRI, typically acquired on the day of treatment, is co-registered to a reference CBCT image set acquired using an on-board CBCT for stereotactic definition. Frame-based treatments can also be defined in a stereotactic manner via conventional fiducial-based methods using MRI and/or CT. This on-board image registration technique was not required for the purposes of this planning study.

For frame-based immobilization, patient set up can be verified using on-board CBCT imaging prior treatment, however, this is not strictly necessary since the frame will not have been removed between the planning CBCT and treatment. If mask-cased immobilization is used then patient set-up is verified using on board CBCT imaging prior to treatment, in addition to intra fraction motion monitoring for the duration of treatment using a high-definition motion management system.^35–38^ Similarly, immobilization was not required, as part of this *in silico* study.

To deliver the dose to a lesion the patient is moved about this focus point to the planned stereotactic co-ordinates by means of a stereotactic couch, with an accuracy of 0.1 mm in *x*, *y*, and *z* directions and a repeatability of 0.05 mm^39^ and is also known as a Patient Positioning System (PPS). Dose to a lesion can be delivered with a single shot (corresponding to a single isocentre PPS coordinate) or multiple shots (multiple isocentre PPS co-ordinates).

For all modalities, treatment plans were optimized in accordance with the NHS England intracranial service specifications.

### Plan quality

Clinical plan quality was assessed by evaluating the overall plan and using dosimetric indices. Dose conformity was evaluated using the Inverse Paddick Conformity Index (CI) which is a defined as follows:^39^

$$CI=\frac{TV\;\times \;\mathrm{PIV}}{{{TV}_{\mathrm{PIV}}}^{2}}$$

where TV is the target volume, PIV is the prescription isodose volume, and TV_PIV_ is the volume of the target covered by the prescription isodose. A plan which is perfectly conformed to the target would have a CI of 1 with less conformal plans having a value of > 1.

The GI, proposed by Paddick^40^ and used in this study, is defined in the following way:

$$GI=\frac{V_{50\%Px}}{V_{100\%Px}}$$

where V_50%Px_ is the volume of half the prescription isodose and V_100%Px_ is the volume of the prescription isodose. The GI is used to measure the dose falloff outside the target and can be used to demonstrate the plan quality, whereby the steepest dose falloff is achieved. The lower the GI the greater the dose falloff and therefore improved dose conformity outside of the target volume.

All plans were assessed by a Medical Physics Expert, each with more than 10 years planning experience. As part of the plan quality evaluation of this study if two or more targets were so proximal that it was not possible to calculate the CI or GI, due to overlapping isodose lines, the targets were excluded from the analysis. Thus, only targets where the true CI and GI could be calculated were included.

Additionally, the minimum and maximum point dose to the targets was evaluated. While a point dose is arguably less clinically relevant than including, for example, the maximum dose to the structure as D0.035 cc,^41^ the point doses do give an indication of both the coverage of the prescription isodose and the heterogeneity of the plan. Since the majority of the lesions were treated to 24 Gy, only the lesions prescribed to this dose were assessed. This gave an indication of how closely the prescription dose was achieved for the plans. A dosimetric constraint of 99.9% target coverage of the prescription isodose was applied for the linac with HD MLCs and 98% for the linac with standard width MLCs and CK in accordance with local planning techniques. While the GK optimizer cannot apply a percentage target coverage constraint, planners aimed to achieve 100% target coverage of the prescription isodose. For all modalities a lower coverage was accepted when the required coverage could not be achieved due to proximity of OARs or target size.

Four of the 62 targets were larger than 3 cm^3^. To ensure the plan quality results were not biased by the larger lesions, given that the largest GK collimator is 16 mm, these targets were excluded from the statistical analysis of CI, GI, minimum, and maximum. Additionally, it should be noted that treating lesions of 0.01 cm^3^ with a linac equipped with 5 mm MLCs may not provide an optimal plan as the leaf width is comparable, if not larger, than the target size. This planning study was expected to reveal the limitations of the 4 treatment modalities considered for multiple brain metastases by the plan quality results.

The total number of MUs required to deliver the prescribed dose by the linac planning techniques were compared. It is preferable to minimize the number of MUs, since the reduced low dose wash will decrease the theoretical risk of secondary malignancies^42^ and shorten overall treatment time.

Finally, the maximum dose to each OAR structure was considered, except normal whole brain, where the volume of brain irradiated by the 12 Gy isodose line and the cochlea where the dose to 50% of the structure was reported. In addition, the dose delivered to 1 cm^3^ of the brainstem was compared. The following dosimetric constraints were applied to the OARs: D50% equal to 4 Gy and maximum dose (D_Max_) of 12 Gy to the cochlea; D1cm^3^ equal to 12 Gy and a D_Max_ equal to 12.5 Gy to the brainstem and D0.2 cm^3^ equal to 8 Gy and a D_Max_ of 10 Gy to the optic apparatus in accordance with the following data.^41^^,^^43–45^ No dose constraint was applied to the cerebella and where possible, for single fraction treatments, the dose to the normal whole brain V12Gy was kept within 10 cm^3^, to minimize the risk of brain radionecrosis.^29^ The principle of ensuring exposure was kept as low as reasonably practicable^46^ was applied across all modalities.

## Results

### Comparison of linacs using HD and standard width MLCs

The CI and GI were assessed for each plan and the results comparing a linac using HD and standard MLCs can be seen in Table 2(a). The linacs with the HD MLCs and standard MLCs were shown to deliver CI values with respective mean values of 1.65 ± 0.36 and 1.67 ± 0.60. This difference was not shown to be statistically significant (*P*-value = .713). The GI for the HD MLCs and standard MLCs gave a respective mean value of 4.89 ± 1.47 and 7.47 ± 2.78, which was a statistically significant difference (*P*-value <.001).

**Table 2.** Comparison of CI, GI, Max, Min between HD MLC and (a) standard MLC linacs, (b) CK and (c) GK. (d) Total number of MUs, total number of arcs and distance from the target centre for HD and standard MLC linacs.

(a)								
	CI		GI		Min (Gy)		Max (Gy)	
	HD MLC	MLC	HD MLC	MLC	HD MLC	MLC	HD MLC	MLC
	1.65 ± 0.36	1.67 ± 0.60	4.89 ± 1.47	7.47 ± 2.78	23.80 ± 1.36	22.66 ± 0.91	32.98 ± 2.84	29.87 ± 2.03
	1.58	1.46	4.72	7.09	24.04	22.69	31.90	29.51
	1.19-3.10	1.20-4.75	2.60-8.08	3.05-14.93	17.78-26.02	19.45-24.49	28.74-41.78	26.62-39.67
	.713		*<.001*		*<.001*		*<.001*	

(b)								
	CI		GI		Min (Gy)		Max (Gy)	
	HD MLC	CK	HD MLC	CK	HD MLC	CK	HD MLC	CK
	1.57 ± 0.24	1.33 ± 0.25	4.75 ± 1.45	6.96 ± 2.75	23.80 ± 1.36	24.01 ± 0.53	32.98 ± 2.84	35.37 ± 6.06
	1.56	1.28	4.54	5.94	24.04	24.14	31.9	33.69
	1.19-2.29	1.00-2.00	2.60-8.08	3.00-14.00	17.78-26.02	21.64-24.96	28.74-41.78	27.37-49.57
	*<.001*		*<.001*		.279		*.029*	

(c)								
	CI		GI		Min (Gy)		Max (Gy)	
	HD MLC	GK	HD MLC	GK	HD MLC	GK	HD MLC	GK
	1.60 ± 0.30	1.86 ± 0.57	5.01 ± 1.54	4.00 ± 0.77	23.80 ± 1.36	21.50 ± 1.88	32.98 ± 2.84	38.25 ± 7.00
	1.57	1.77	4.73	3.78	24.04	21.95	31.9	36.90
	1.19-2.75	1.11-3.42	2.60-8.08	2.84-5.81	17.78-26.02	15.00-24.40	28.74-41.78	27.10-50.00
	*.005*		*<.001*		*<.001*		*<.001*	

(d)						
	Total MUs		Total number of arcs		Distance to isocenter (mm)	
	HD MLC	MLC	HD MLC	MLC	HD MLC	MLC
	16462 ± 5421	9299 ± 2146	10.5 ± 3.3	11.4 ± 2.2	18.4 ± 12.7	40.9 ± 20.3
	15795	8974	11	10	20	39
	8692 - 30722	5924 - 12544	5–17	10–15	0 - 45	0 - 92
	*<.001*		.574		*<.001*	

Abbreviations: CI = conformity index, GI = gradient index, Min = minimum point dose, Max = maximum point dose, HD MLC = linac using high-definition MLCs, MLC = linac using standard MLCs, MU = monitor units, CK = CyberKnife, GK = Gamma Knife.

Table 2(a) shows the minimum and maximum point doses to the lesions prescribed to 24 Gy comparing a linac using HD and standard MLCs. The minimum point dose to the target using the linac with the HD MLCs and the standard MLCs gave respective mean values of 23.80 ± 1.36 and 22.66 ± 0.91. The null hypothesis was rejected (*P*-value <.001), indicating that HD MLCs achieved a result closer to the prescribed 24 Gy than standard MLCs, with a median minimum point dose of 24.04 Gy. The maximum point dose to the targets using a linac with HD and standard MLCs gave mean values of 32.98 ± 2.84 and 29.87 ± 2.03, respectively, a statistically significant difference (*P*-value <.001). A higher maximum point dose to targets is expected with the use of HD MLCs, due to the contribution of multiple plans, whereas single plans were used for the standard MLCs.

The total number of MUs for the linac using HD MLC versus standard MLC plans are summarized in Table 2(d). The mean total number of MUs is 44% lower using standard MLC compared to the HD MLC plans, a statistically significant result (*P*-value <.001). The use of HD MLCs results in a higher number of MUs as multiple plans were required to ensure all the targets were treated with the 2.5 mm MLCs, due to the distance of the targets from the isocentre. A single plan was achievable using standard MLCs, resulting in a lower number of MUs.

A comparison of the total number of arcs and distance from the centre of the targets to the plan isocentre is reported in Table 2(d). The total number of arcs for HD MLC compared to standard MLC plans was 10.5 ± 3.3 and 11.4 ± 2.2, respectively, a non-significant difference (*P*-value = .574).

The mean distance of targets from isocentre was significantly less using the HD MLCs (18.4 ± 12.7 mm) than with the standard MLC plans (40.9 ± 20.3 mm), *P*-value <.001. This is because the HD MLCs extend to a field size of 10 × 10 cm^2^. Therefore, to ensure that all targets were covered by HD MLCs the distance from plan isocentre was always <50 mm. Such a restriction did not exist with the standard MLCs, resulting in a mean distance from isocentre which was more than double that of HD MLC plans.

Table 3(a) summarizes the dosimetric results for OARs. For all OARs the dose was lower using HD MLCs over standard MLCs, with the exception of hippocampal dose. As shown in Table 3(a), half of the OAR comparison criteria demonstrated a statistically significant difference: maximum dose to brainstem (*P*-value = .042), maximum dose to optic nerves (*P*-value = .032), maximum dose to optic tracts (*P*-value = .005), and normal whole brain V12Gy (*P*-value = .049). For all statistically significant OAR results, HD MLCs achieve an improved dose sparing over standard MLCs.

**Table 3.** Comparison of OAR doses for (a) HD and standard MLC linacs (b) HD MLC and CK and (c) HD MLC and GK.

(a)											
		Brainstem									
		Max dose (Gy)	1cc (Gy)	Normal Cer max (Gy)	Chiasm max (Gy)	Cochlea mean (Gy)	Eye max (Gy)	Normal Hipp max (Gy)	Optic Nerve max (Gy)	Optic Tract max (Gy)	Normal WB V12Gy (cc)
**HD MLCs**		5.14 ± 3.87	3.13 ± 2.44	23.83 ± 11.26	1.64 ± 1.66	2.10 ± 1.99	1.19 ± 0.31	5.03 ± 6.90	1.36 ± 0.78	2.23 ± 2.19	12.41 ± 10.04
		2.74	1.84	27.32	1.08	1.49	1.11	3.14	1.18	1.46	7.80
		1.17-12.03	1.00-8.86	1.95-39.19	0.49-6.70	0.38-7.40	0.54-1.62	1.40-26.60	0.49-3.56	0.68-8.86	0.73-33.79
**MLCs**		6.19 ± 4.03	3.83 ± 1.96	24.21 ± 11.86	2.26 ± 0.95	2.79 ± 2.80	1.48 ± 0.59	4.75 ± 4.91	1.90 ± 0.64	3.10 ± 2.14	14.48 ± 11.38
		5.61	3.95	29.65	2.22	1.87	1.46	3.35	1.86	2.79	9.41
		2.05-14.09	1.5-8.16	2.53-41.54	0.85-4.71	0.40-11.07	0.38-2.79	1.66-19.77	0.50-2.79	1.08-9.34	2.50-36.90
		*.042*	.054	.638	.054	.175	.148	.577	*.032*	*.005*	*.049*

(b)											
		Brainstem									
		Max dose (Gy)	1cc (Gy)	Normal cer max (Gy)	Chiasm max (Gy)	Cochlea mean (Gy)	Eye max (Gy)	Normal hipp max (Gy)	Optic Nerve max (Gy)	Optic Tract max (Gy)	Normal WB V12Gy (cc)
**HD MLCs**		5.14 ± 3.87	3.13 ± 2.44	23.83 ± 11.26	1.64 ± 1.66	2.10 ± 1.99	1.19 ± 0.31	5.03 ± 6.90	1.36 ± 0.78	2.23 ± 2.19	12.41 ± 10.04
		2.74	1.84	27.32	1.08	1.49	1.11	3.14	1.18	1.46	7.80
		1.17-12.03	1.00-8.86	1.95-39.19	0.49-6.70	0.38-7.40	0.54-1.62	1.40-26.60	0.49-3.56	0.68-8.86	0.73-33.79
**CK**		5.87 ± 4.31	3.98 ± 3.18	20.78 ± 11.16	2.46 ± 1.83	1.75 ± 1.82	0.41 ± 0.19	6.62 ± 7.02	2.09 ± 1.66	3.32 ± 2.19	11.98 ± 10.83
		3.29	1.98	25.46	2.03	0.73	0.40	4.10	2.35	2.54	6.88
		0.67-13.18	0.53-8.72	3.54-35.48	0.19-5.27	0.19-5.46	0.11-0.67	1.78-27.29	0.16-4.66	0.40-7.54	1.94-32.71
		.102	.320	.206	.465	.520	*.001*	*.024*	.365	.083	.846

(c)											
		Brainstem									
		Max dose (Gy)	1cc (Gy)	Normal cer max (Gy)	Chiasm max (Gy)	Cochlea mean (Gy)	Eye max (Gy)	Normal hipp max (Gy)	Optic Nerve max (Gy)	Optic Tract max (Gy)	Normal WB V12Gy (cc)
**HD MLCs**		5.14 ± 3.87	3.13 ± 2.44	23.83 ± 11.26	1.64 ± 1.66	2.10 ± 1.99	1.19 ± 0.31	5.03 ± 6.90	1.36 ± 0.78	2.23 ± 2.19	12.41 ± 10.04
		2.74	1.84	27.32	1.08	1.49	1.11	3.14	1.18	1.46	7.80
		1.17-12.03	1.00-8.86	1.95-39.19	0.49-6.70	0.38-7.40	0.54-1.62	1.40-26.60	0.49-3.56	0.68-8.86	0.73-33.79
**GK**		4.92 ± 4.41	2.81 ± 2.21	30.85 ± 17.02	1.15 ± 0.71	1.53 ± 1.83	1.24 ± 0.88	5.71 ± 12.07	1.13 ± 0.48	1.89 ± 1.67	8.65 ± 6.40
		4.20	3.00	36.20	0.80	0.60	1.00	1.80	1.10	1.10	5.48
		0.70-16.00	0.40-7.30	0.70-48.10	0.30-2.50	0.10-5.70	0.20-3.40	0.80-43.70	0.20-1.90	0.60-5.10	1.18-18.61
		.320	.465	*.042*	.413	*.024*	.898	.123	.413	.320	*.014*

Abbreviations: Max = maximum, Cer = cerebellum, D50 = dose to 50% of the structure, Hipp = hippocampus, WB = whole brain, V12Gy = volume of structure encompassed by the 12 Gy isodose line, HD MLC = isocentric linear accelerator using high-definition MLCs, MLC = isocentric linear accelerator using standard MLCs, CK = CyberKnife, GK = Gamma Knife.

### Comparison of HD MLC linac and CK

The following results compare a linac with HD MLCs and CK plans. As with the previous results, only the targets whose isodoses do not overlap with proximal lesions were analysed. Therefore, only data with distinguishable targets from both modalities were evaluated and this differs slightly with each compared dataset.

Comparing the CI for plans generated using the linac with HD MLCs and CK gave significantly different mean values of 1.55 ± 0.25 and 1.32 ± 0.24, respectively (*P*-value <.001), as shown in Table 2(b). The mean GI for the linac with HD MLCs was 4.61 ± 1.47, while CK delivered a mean GI of 6.69 ± 2.82, a statistically significant difference (*P*-value <0.001). Therefore, whilst plan conformity was better with CK, dose fall off was improved using the linac with HD MLCs.

A summary of minimum and maximum point doses to targets prescribed to 24 Gy for the linac with HD MLCs and CK are shown in Table 2(b). Both treatment modalities achieved a comparable minimum point dose, close to the prescribed dose, with a median value of 24.02 and 24.13 Gy for the linac with HD MLCs and CK, respectively (*P*-value = .196). However, the mean maximum point dose to targets was significantly higher (*P*-value = .020) using CK (35.46 ± 6.04 Gy) compared to the linac with HD MLCs (33.01 ± 2.81 Gy).

Table 3(b) shows OAR doses for the linac with HD MLCs and CK plans. For the majority of the OAR dose constraints there is no statistically significant difference between the treatment modalities. The exceptions were the maximum dose to the eye (*P*-value = .001) and normal hippocampus (*P*-value = .024). The mean maximum dose to the eye was lower using CK (0.41 ± 0.31 Gy) than the linac using HD MLCs (1.19 ± 0.31 Gy), while the mean maximum dose to the normal hippocampus was lower using the linac with HD MLCs (5.03 ± 6.90 Gy) than with CK (6.62 ± 7.02 Gy).

### Comparison of linac with HD MLCs and GK

The following results summarize a plan quality comparison of the linac with HD MLCs and GK. As with the previous results, proximal targets which precluded a calculation of the CI and GI due to overlapping isodoses were not included.

Dosimetric indices for the linac with HD MLCs and GK are shown in Table 2(c). There is a statistically significant improvement (*P*-value = 1.162 × 10^−2^) in mean CI using the linac with HD MLC (1.56 ± 0.30) versus GK (1.79 ± 0.58). The dose fall off, however, was significantly improved with GK (*P*-value <.001), delivering a mean GI of 3.87 ± 0.81 compared to 4.78 ± 1.60 for the linac with HD MLC plans.

Evaluating the doses for targets prescribed to 24 Gy, Table 2(c), it was shown that the linac with HD MLC delivers a minimum point dose closer to the prescription than GK with respective median point doses of 24.02 and 21.90 Gy and this difference was statistically significant (*P*-value <.001). The maximum point dose to the targets was also significantly lower (*P*-value <.001) using the linac with HD MLC than with GK with a median dose of 31.91 and 36.90 Gy, respectively.

Results for OARs, Table 3(c), demonstrate a statistically significant dose reduction (*P*-value = .042) using the linac with HD MLCs for maximum normal cerebellum, while for mean cochlea and normal whole brain V12Gy, GK delivered a statistically significant dose reduction (*P*-values of .024 and .014, respectively).

### Dose distribution and DVH comparison

**Figure 2.**
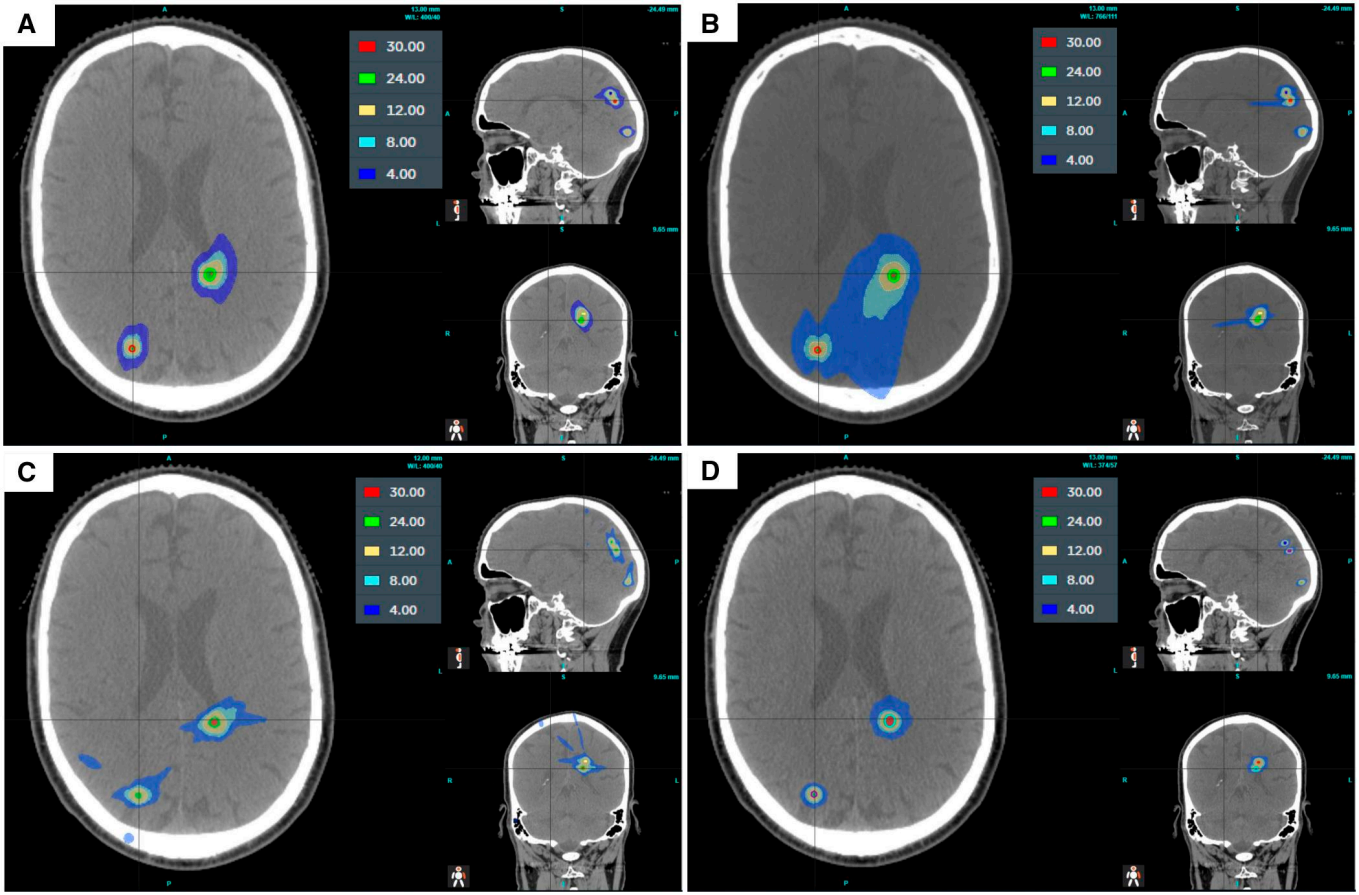
Dose distribution for (A) linac with HD MLCs and (B) standard MLCs; (C) CK and (D) GK for a patient with 5 targets all treated to 24 Gy.

The dose distribution of the four treatment modalities is shown in Figure 2 for a patient with five metastatic lesions all prescribed to 24 Gy (patient 1 in Table 1). The low dose distribution is lower for the linac with HD MLCs and GK than with standard MLCs or CK. Hotspots are larger for CK and GK because these treatment techniques percentage isodose that the lesion is prescribed to may be lower than with the linac-based treatments and not expected to be clinically deleterious.

**Figure 3.**
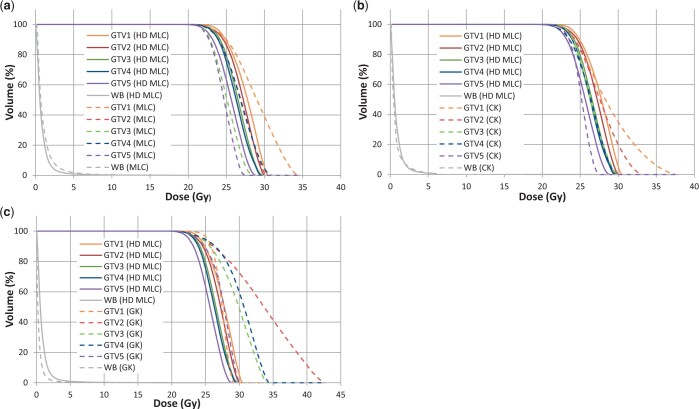
Cumulative DVHs of the five PTVs all prescribed to 24 Gy and the normal whole brain for a selected patient comparing the linac using HD MLCs with (A) standard MLCs, (B) CK, and (C) GK. Abbreviations: WB = normal whole brain, DVH = dose-volume histogram, HD MLC = linac using high-definition MLCs; MLC = linac using standard MLCs, CK = CyberKnife, GK = Gamma Knife.

Figure 3 reports the cumulative dose-volume histograms (DVHs) for the patient shown in Figure 2 (patient 1 in Table 1) with the 5 malignancies prescribed to 24 Gy and compares the linac using HD MLCs with standard MLCs, CK, and GK. The DVH for the 5 lesions and the normal whole brain is included. For each DVH, the structures for the linac using HD MLC plans are represented by the solid lines while the other treatment modalities are shown as dashed lines.

In Figure 3A, GTV1, GTV2, and GTV4 have steeper curves for the linac with HD MLCs suggesting greater dose homogeneity within the target. There is a notable improvement in the normal whole brain dose sparing for the linac using HD MLCs, consistent with the results of Table 3(a).

Comparing the DVHs for the linac with HD MLCs and CK, as shown in Figure 3B, it can be observed that the maximum dose for the targets was higher for CK than the linac with HD MLCs, in agreement with Table 2(b). The dose sparing of normal whole brain is similar for both modalities for this patient, which supports the results of Table 3(b).

Figure 3C compares the DVHs for the linac with HD MLCs with GK. As with CK, GK in general delivers a higher dose to the targets than the linac, however, GK provides a greater dose sparing to normal whole brain than any other modality for this patient and is consistent with the results in Table 3(c).

## Discussion

This multi-centre *in silico* planning study has demonstrated an improvement in the dose falloff using HD MLCs compared with standard MLCs for a linac used for SRS in the treatment of multiple brain metastases. Plans for the linac with standard MLCs used a single isocentre technique, while for HD MLC plans, a combination of a single isocentre for multiple lesions and a single isocentre per lesion was utilized, where appropriate, to ensure all the targets were treated within fields defined by the HD (2.5 mm width) MLCs.

Evaluating the doses to the plans prescribed to 24 Gy, the linac with HD MLCs more closely met the minimum point prescribed dose but gave a higher maximum point dose to targets, compared to the standard MLC based plans. A significantly lower OAR dose was achieved for 4 of the 10 evaluation criteria using the linac with HD MLCs compared to standard MLCs.

When using linacs to treat multiple targets, rotational errors are increased with distance from isocentre. By simulating a rotational error of 1° about the plan isocentre, applied uniformly in all 3 axes, it has been shown that 7% of targets had D95% and V95% coverage of less than 95% for single-isocentre multiple target SRS plans.^10^ In another study, a collimator rotation error of 1° resulted in unacceptable dosimetric changes to the V100% and D99% for 5% and 6% of targets, respectively for multiple target single isocentre plans, while multiple isocentre plans were relatively unchanged.^42^ For the linac plans in this study, it was shown that there was a statistically significant reduction in the mean target distance to isocentre with HD MLC (*P*-value <.001) when compared to the standard MLC plans. A lower mean target distance to isocentre will result in a reduction in the rotational geometric errors introduced, however, it would be possible to reduce such errors on linacs with standard MLCs by limiting the target distance from isocentre.^47^

There was no difference in the total mean number of arcs used when comparing the linac using HD and standard MLCs, which would enable the plans to be delivered in a similar timeframe. There will, however, be an increase in imaging and set-up time when using multiple isocentres.

While the linac with HD MLCs offers clear advantages over the standard MLCs, the results comparing the linac using HD MLCs with CK demonstrated plan quality strengths with both modalities. CK delivered significant improvements in CI, while dose fall off was significantly improved using the linac with HD MLCs. Significant differences in the minimum point prescription doses were not found for either modality, while maximum point dose to the target was significantly higher using CK. OAR doses revealed no significant difference; with two constraints significantly lower using CK and one constraint significantly lower using the linac with HD MLCs. The significant reduction in eye dose using CK was because the beams entering the eye are blocked with a 10 mm margin.

Again, minor strengths in plan quality for both modalities were found when the linac with HD MLCs was compared to GK. CI was significantly improved using the linac with HD MLCs, while GK gave a significantly lower GI. The minimum and maximum point doses were significantly lower and higher, respectively, using GK. The linac using HD MLCs delivering significantly lower OAR dose sparing for one OAR constraint and GK giving significantly lower dose for two OAR criteria. Importantly, the volume of normal whole brain receiving 12 Gy was significantly lower with GK. Typically, radiation necrosis of the brain is expected to occur between 3 months and several years (median 1-2 years). The V12Gy constraint to the normal whole brain is expected to reduce the risk of necrosis to < 20%.^48^

Finally, the dose distribution and DVH analysis of a typical patient with 5 metastases echoed the comparative results of the treatment modalities, with GK demonstrating the lowest GI, lowest normal whole brain dose and highest maximum target doses of all the treatment types, while the linac with HD MLCs showed improved results when compared to standard MLC plans not a statistically significant difference to CK.

While there are clear plan quality advantages offered by HD MLCs versus standard MLC linacs, the results of this study demonstrate little superiority in plan quality when comparing HD MLCs with CK or GK for treating multiple brain metastases, in agreement with other studies.^14^^,^^16^^,^^49^ However, this study goes further by combining both VMAT single isocentre and DCA multiple isocentre HD MLC linac-based plans for comparison with a standard width MLC linac, CK and GK. This study also examines the effects of using both a single and multiple isocentric planning technique has on the distance from isocentre and the number of MUs required and considers the impact on the geometric errors introduced.

The appropriate selection of modality must be considered, particularly when considering challenging multiple brain metastases cases. For example, linacs with MLCs are associated with leaf position uncertainties,^50^ while GK and CK use fixed size collimators. Unlike frame-based treatments, frameless SRS affect patient immobilization required the introduction of margins.^51^ Image registration errors may also affect the spatial dose delivery accuracy,^52–54^ however, this consideration was beyond the scope of this study since the same structures were used when planning with each modality. Therefore, as well as plan quality, it is important to consider all relevant factors when selecting a platform to treat a given case.

A limitation of this study was that different planning systems were used to analyse the dose to structures. Since each planning system models structures slightly differently, this may result in very small (<0.1 cm^3^) differences in volumes. It should also be considered that plan quality becomes more difficult to assess with very small lesions. Dose statistics are poorer for very small targets, without necessarily resulting in inferior clinical outcomes. Finally, for this cohort of patients, differences in the prescriptions and target margins would have existed across the centres and modalities. Typically, a 1 mm margin is added to the GTV to create a PTV for linac plans, while no margin is added to CK or GK targets.^51^^,^^55^ However, prescriptions and margins were kept the same in this study, to allow a direct comparison.

A further limitation of this study, which is arguably true for all planning studies with multiple planners, is the influence of the planner’s expertise and experience. All plans were checked by Medical Physics Experts each with more than 10 years of radiotherapy planning experience, respectively. Furthermore, a recent study compared the plan quality of one of the TPSs used in this study with other TPSs and demonstrated that high and consistent plan quality was achievable using the automated TPS independent of the planner’s experience for planning SRS to intracranial metastases.^56^ Additionally, the NHS England intracranial service specifications for plan quality acceptability were adopted as the guiding principle for minimum plan quality. A larger patient cohort may also be expected to more closely reveal the differences in the plan quality as a result of the treatment modalities, rather than the skills and expertise of the planner.

## Conclusion

For linacs, HD MLCs are the preferred choice for delivering SRS to multiple brain metastases, providing superior target coverage and OAR dose sparing, when compared to standard MLCs, without adversely affecting treatment times. The linac with HD MLCs, CK, or GK, were shown to offer significant improvements in different aspects of plan quality. These results are expected to guide radiotherapy departments intending to purchase new stereotactic equipment without compromising on treatment delivery.
